# Radiomics for Predicting the Efficacy of Immunotherapy in Hepatocellular Carcinoma: A Systematic Review and Radiomics Quality Score Assessment

**DOI:** 10.3390/cancers18020186

**Published:** 2026-01-06

**Authors:** Ruixin Zhang, Chengjie Zhang, Yi Liu, Zhiguo Gui, Anhong Zhang

**Affiliations:** 1 First Clinical Medical College, Shanxi Medical University, Taiyuan 030001, China; zhangruixin@sxmu.edu.cn (R.Z.); zhangchengjie1@sxmu.edu.cn (C.Z.); 2 School of Information and Communication Engineering, North University of China, Taiyuan 030051, China; liuyi@nuc.edu.cn; 3 State Key Laboratory of Extreme Environment Optoelectronic Dynamic Testing Technology and Instrument, North University of China, Taiyuan 030051, China; 4 Department of Hepatobiliary Surgery and Liver Transplantation Center, First Hospital of Shanxi Medical University, Taiyuan 030001, China

**Keywords:** hepatocellular carcinoma, immunotherapy, radiomics, treatment outcome, radiomics quality scoring, systematic review

## Abstract

Background/Objectives: Hepatocellular carcinoma (HCC) immunotherapy provides limited clinical benefits, partly due to the lack of reliable efficacy biomarkers. Radiomics, which non-invasively analyzes tumor heterogeneity, shows promising potential for predicting treatment outcomes. Methods: The present study systematically evaluated the predictive performance and methodological quality of radiomics models for assessing immunotherapy efficacy in patients with HCC. A literature search was conducted in PubMed, Web of Science, Embase, and the Cochrane Library for studies published up to 21 June 2025, which developed CT- or MRI-based radiomics models to predict immunotherapy efficacy in HCC. Study quality was assessed using the radiomics quality score (RQS) and the METhodological RadiomICs Score (METRICS). Results: A total of 11 studies were included and categorized by immunotherapy regimen: ICIs alone (1/11), ICIs combined with targeted therapy (6/11), and ICIs combined with targeted therapy plus locoregional therapy (4/11). The models primarily predicted treatment response (7/11), overall survival (OS) (4/11), or progression-free survival (PFS) (4/11). In the ICI monotherapy cohort, AUC values for predicting treatment response ranged from 0.705 to 0.772. In the ICI plus targeted therapy cohorts, AUC or concordance index (C-index) values for predicting the above efficacy endpoints were 0.792–0.956, 0.63–0.77, and 0.54–0.837, respectively. In the combination therapy cohorts incorporating locoregional treatment, predictive models showed AUC or C-index values of 0.721–0.92, 0.817–0.838, and 0.59. Quality assessment revealed a median RQS of 15 (range: 11–19) and a median METRICS of 72.5% (range: 56.0–79.5%) across all studies. Conclusions: CT/MRI-based radiomics uses routine imaging to non-invasively quantify whole-tumor phenotype and heterogeneity, enabling repeatable, longitudinal assessment in hepatocellular carcinoma. Evidence suggests that it can help to identify patients likely to benefit from immunotherapy before treatment. However, clinical implementation requires standardized imaging and analysis protocols, external validation, and transparent reporting.

## Simple Summary

Radiomics shows strong potential to predict immunotherapy efficacy in hepatocellular carcinoma, whether used alone or with immune checkpoint inhibitors. Current models perform better for short-term responses (mRECIST/RECIST 1.1) than for long-term outcomes (overall survival/progression-free survival). Integrating radiomic features with clinical characteristics markedly improves prediction. Major challenges persist: heterogeneous imaging and protocols, limited external generalizability, weak biological interpretability, suboptimal clinical applicability, and poor data sharing. This review synthesizes current evidence and recommends prioritizing standardization, multimodal and clinical data fusion, prospective multicenter validation, and the adoption of open, FAIR-compliant datasets to facilitate the translation of radiomics into reliable decision-support tools for personalized immunotherapy in hepatocellular carcinoma.

## 1. Introduction

Primary liver cancer (PLC) is the sixth most common malignancy worldwide and the third leading cause of cancer-related death [1]. Hepatocellular carcinoma (HCC), the main histological subtype of PLC, accounts for about 80% of cases [2]. Due to its insidious onset, most patients with HCC are diagnosed at advanced stages, when curative surgical resection is no longer possible. Systemic therapies, particularly immune checkpoint inhibitors (ICIs), have become central to the treatment of unresectable HCC [3,4]. In 2017, nivolumab was approved by the FDA as the first second-line therapy for HCC, marking the start of the immunotherapy era [5]. In July 2019, the FDA granted breakthrough therapy designation to the combination of the PD-1 inhibitor pembrolizumab with lenvatinib as a first-line treatment for unresectable HCC [6]. Since then, combined approaches that integrate locoregional and systemic therapies have increasingly been utilized in clinical practice [7]. Nonetheless, due to the marked biological heterogeneity of HCC, only a subset of patients responds favorably to immunotherapy. Studies have shown that with ICI monotherapy, durable objective responses are achieved in only 15–20% of patients [8]. The subsequent development of various combination strategies, such as ICIs with molecularly targeted agents, and further integration with locoregional treatments, has improved response rates and survival outcomes in patients with HCC. Nevertheless, the therapeutic benefit of these regimens is still confined to a limited proportion of patients [9]. Although multiple immunotherapy combinations are now available, non-responders face substantial challenges, including increased medical costs, a higher risk of severe adverse events, and the possible loss of the optimal treatment window for other effective therapies. Therefore, accurately identifying patients who are most likely to benefit from specific regimens is crucial for advancing precision and individualized treatment.

Currently, no reliable biomarkers are available to accurately predict the efficacy of immunotherapy in patients with HCC. Proposed biomarkers include programmed death-ligand 1 (PD-L1) expression [10], tumor-infiltrating lymphocytes [11], tumor mutation burden (TMB) [12], and the expression of specific genes or signaling pathways. However, these tissue-based biomarkers require invasive biopsy to obtain tumor samples and cannot fully capture the spatial and temporal heterogeneity of tumors. Radiomics, as a new artificial intelligence technology, enables the extraction of high-throughput quantitative features from medical images such as CT and MRI, transforming them into analyzable data for the non-invasive assessment of tumor heterogeneity [13]. Radiomics has already been applied to the differential diagnosis, molecular subtyping, and prognostic evaluation of HCC [14].

Due to marked tumor heterogeneity, the clinical benefit of ICIs in HCC varies substantially among patients, and robust, reproducible, and scalable biomarkers for treatment response remain lacking. Radiomics enables non-invasive, quantitative characterization of tumor phenotypes and has emerged as a promising approach for predicting therapeutic responses. However, current radiomics research faces challenges in reproducibility and generalizability across scanners and institutions, methodological transparency (e.g., incomplete reporting of image acquisition, segmentation, and feature extraction), and biological interpretability, all of which may hinder clinical translation. In recent years, several studies have developed CT- or MRI-based radiomics models to predict the efficacy of immunotherapy in patients with HCC. However, no systematic review has yet been conducted to integrate and evaluate their overall performance and methodological quality. Therefore, this study aims to systematically summarize the available evidence to assess the predictive performance of radiomics models for immunotherapy efficacy in HCC and to evaluate the reporting quality of these studies. Collectively, our findings provide evidence-based support for the clinical translation of radiomics in precision immunotherapy for HCC.

## 2. Methods

**Figure 1:**
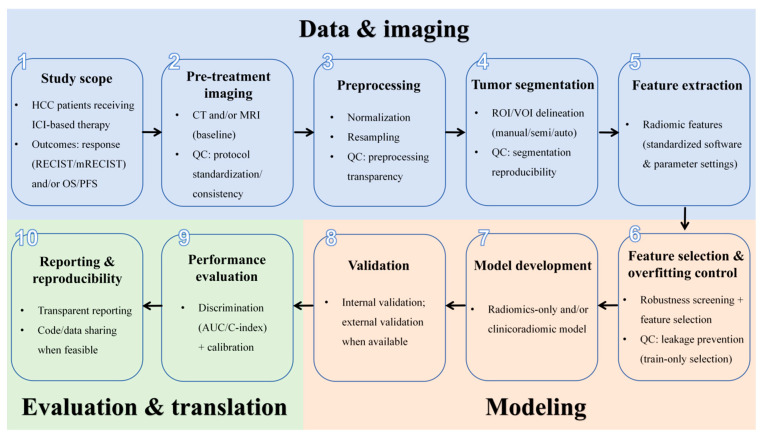
Schematic overview of the radiomics pipeline for immunotherapy outcome prediction in hepatocellular carcinoma (QS, quality score).

This systematic review strictly follows the Preferred Reporting Items for Systematic Reviews and Meta-Analyses (PRISMA) guidelines [15], and the PRISMA 2020 Checklist. We present a schematic overview of a typical CT/MRI-based radiomics pipeline for predicting immunotherapy outcomes in HCC (Figure 1).

### 2.1. Search Strategy

A comprehensive literature search was performed in PubMed, Web of Science, Embase, and the Cochrane Library to identify studies published from database inception to 21 June 2025. The search strategy combined Medical Subject Headings (MeSH) and free-text terms, including “Radiomics”, “Hepatocellular Carcinoma”, and “Immune Checkpoint Inhibitor”. Detailed search strategies for each database are provided in the Supplementary Materials (Supplementary Table S1). In addition, the reference lists of the included articles were screened to identify any other relevant studies.

### 2.2. Study Selection

All retrieved studies were imported into EndNote version X9 for management. After removing duplicates, two independent researchers screened the titles and abstracts to preliminarily exclude irrelevant articles. The full texts of the remaining studies were then obtained and reviewed according to predefined inclusion and exclusion criteria. Any disagreements during the screening process were resolved through consultation with the corresponding author. Given the high dimensionality and multi-step nature of radiomics modeling, extremely small cohorts are highly susceptible to overfitting and feature instability and frequently lack robust internal and external validation. To mitigate small-sample biases and prevent overly optimistic performance estimates, we pre-specified a minimum sample size (*n* ≥ 50).

**Figure 2:**
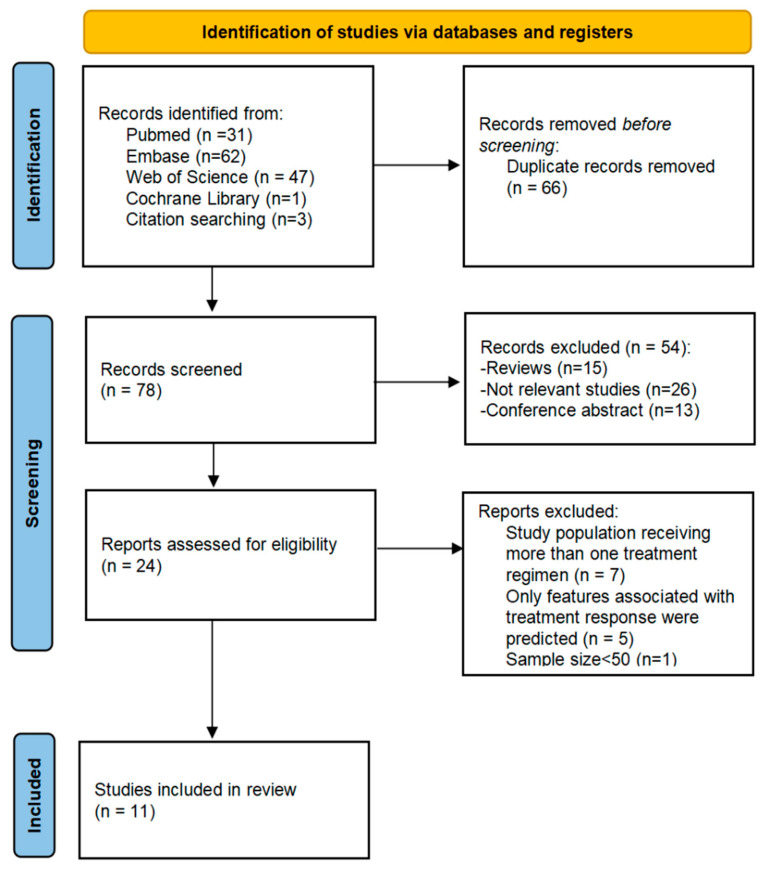
The PRISMA flowchart of study selection.

Eligibility criteria and the selection process are summarized in the PRISMA flow chart (Figure 2). We included original studies involving histologically or clinically confirmed HCC patients treated with ICI-based therapy that utilized pretreatment CT or MRI radiomics to predict immunotherapy outcomes. We excluded studies focusing exclusively on molecular or radiogenomic features rather than clinical efficacy endpoints.

### 2.3. Data Extraction

The following information was extracted from the original studies: (1) Study characteristics: first author, country, publication year, study design, and sample size; (2) Patient characteristics: age, gender, treatment regimen received, and predicted outcome indicators; (3) Radiomics process characteristics: imaging modality, region of interest (ROI) segmentation method, dimension reduction techniques for radiomic features, and algorithms used to construct the model; and (4) Predictive performance indicators of radiomics models: area under the curve (AUC) and concordance index (C-index). In cases where multiple models were constructed in a study, the model with the highest AUC or C-index value from the validation cohort was selected. Given the significant heterogeneity in the design of these studies, this review does not perform a meta-analysis to summarize AUC, C-index, and other indicators. Instead, this study provides a categorized description based on the type of immune-combination therapy and the different efficacy evaluation metrics.

### 2.4. Quality Assessment

For the research quality assessment, two tools were employed: the radiomics quality score (RQS) (https://www.radiomics.world/rqs, accessed on 22 June 2025) and the METhodological Radiomics Quality Score (METRICS) (https://metricsscore.github.io/metrics/METRICS.html, accessed on 22 June 2025). The RQS, proposed by Lambin et al. in 2017, consists of 16 items with a total score range of 0–36 [13]. It evaluates six dimensions: image protocol, feature extraction, data analysis and statistics, model development and validation, clinical applicability, and open science. The RQS is a classical tool widely used to assess the methodological quality of radiomics models; however, it has limitations in interpretability and applicability for deep learning studies. To address these limitations, the European Society of Medical Imaging Informatics introduced METRICS [16]. This tool includes 30 items (+5 conditional items) across nine categories: study design, imaging data, segmentation, image processing and feature extraction, feature processing, preparation for modeling, metrics and comparison, testing, and open science. Two researchers independently applied RQS and METRICS to evaluate the quality of the included radiomics studies. Each reviewer assigned individual scores, and any discrepancies were resolved through discussion or consultation with senior researchers.

## 3. Results

### 3.1. Literature Search

A total of 144 relevant publications were identified in the initial literature search, with an additional 3 articles obtained from the references of related studies. After removing 66 duplicates, 54 articles were excluded based on title and abstract screening due to irrelevance or because they were conference abstracts or reviews. Full texts of the remaining 24 articles were then reviewed, and 13 were excluded for not meeting the inclusion criteria. Ultimately, 11 studies were included in this review. The study selection process is presented in Figure 2.

### 3.2. Overall Characteristics of Included Studies

This systematic review included 11 studies [17,18,19,20,21,22,23,24,25,26,27], of which 10 (90.9%) were conducted in China. All studies were retrospective and published between 2021 and 2025, with six being multicenter studies. A total of 2014 patients were included, with the majority being male (87.3%) and the average age ranging from 47 to 67 years. Based on the immunotherapy regimens administered to patients, the studies were categorized into three groups: ICI monotherapy (1/11), ICIs combined with molecular targeted agents (6/11), and ICIs combined with molecular targeted agents plus locoregional treatments (4/11). Regarding outcome measures, seven studies focused on predicting treatment response, while four specifically predicted OS and PFS. The characteristics of the included studies are summarized in Table 1.

### 3.3. Methodological Quality Assessment

**Figure 3:**
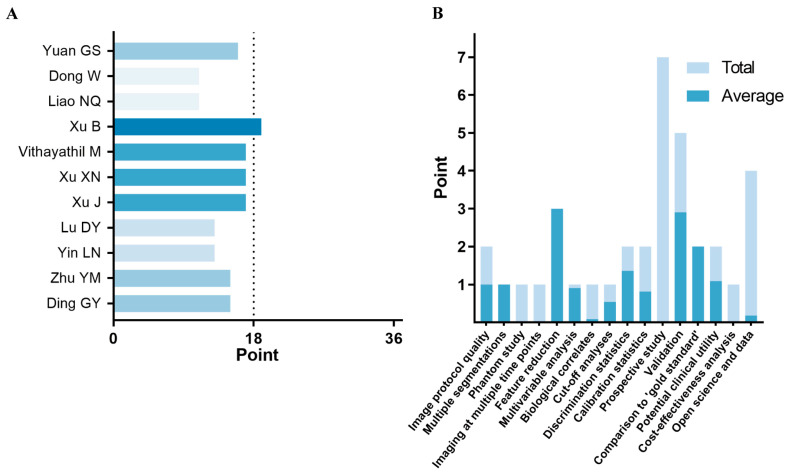
Methodological quality assessment of the studies was performed using radiomics quality score (RQS), including (**A**) RQSs across studies [17,18,19,20,21,22,23,24,25,26,27] and (**B**) the average score for each domain.

The detailed RQS results for each study are presented (Figure 3). The median RQS across all studies was 15 (range: 11–19), corresponding to a median percentage of 41.7% (range: 30.6–52.8%). For specific items, no study scored on the following four items: phantom study, imaging at multiple timepoints, prospective study registered in a trial database, and cost-effectiveness analysis. Only one study examined the correlation between radiomic features and biological characteristics [24]. Conversely, the studies performed well on the following four items: multiple segmentations, feature reduction or adjustment for multiple testing, multivariable analysis with non-radiomics features, and comparison to a gold standard, with average scores exceeding 90%.

**Figure 4:**
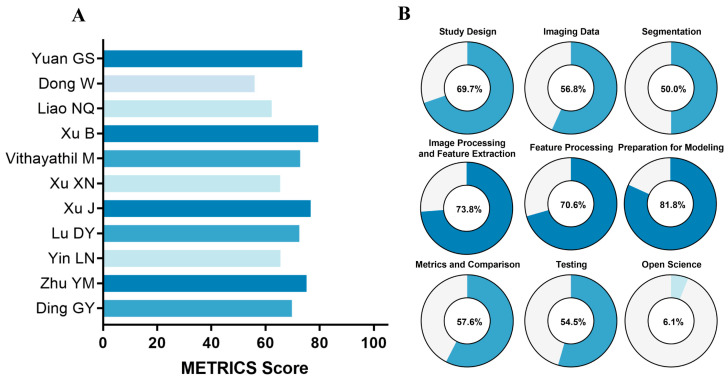
Methodological quality assessment of the studies was performed using METhodological RadiomICs Score (METRICS), including (**A**) METRICSs across studies [17,18,19,20,21,22,23,24,25,26,27] and (**B**) the weighted average score for each domain.

The detailed METRICSs for each study are presented (Figure 4). The median METRICS across all studies was 72.5% (range: 56.0–79.5%). For each dimension, the proportion of “yes” responses was calculated for individual items. The open science dimension had the lowest proportion, at 6.1%, with only two studies providing accessible codes [24,25]. In contrast, the dimensions of image processing and feature extraction, feature processing, and preparation for modeling performed well, with more than 70% of studies answering “yes” for items in these categories. A detailed assessment of each study by item and dimension is provided (Supplementary Tables S2 and S3).

### 3.4. Characteristics of the Radiomics Model Pipeline

The feature extraction parameters and validation methods are summarized in Table 2. The workflow for constructing radiomics models generally involves five main steps: image acquisition, image segmentation, feature extraction, feature selection and dimension reduction, and model development. (1) Image acquisition: Seven studies used CT-derived features to build predictive models, while four studies relied on MRI images. Most studies employed multiphase imaging, with only one study using a single-phase image (portal venous phase, PVP) [23]. Additionally, the majority of studies (7/11) utilized multiple imaging devices for acquisition (Supplementary Table S4). (2) Image segmentation: Ten studies performed ROI segmentation, with seven using manual segmentation and three employing semi-automated methods. (3) Feature extraction: Pyradiomics was the most commonly used extraction tool (7/11). Across the studies, the median number of extracted features was 2236 (range: 428–3376). (4) Feature selection and dimension reduction: Six studies assessed feature reproducibility using the intraclass correlation coefficient (ICC) before feature selection, only retaining features with ICC values above a predefined threshold (minimum 0.75) for further analysis. The least absolute shrinkage and selection operator (LASSO) was the most commonly used method for feature selection. After dimensionality reduction, the median number of retained features was 10 (range: 5–32). (5) Model development: Most studies (8/11) applied more than one algorithm for model construction, with random forest (RF) (4/11) and support vector machine (SVM) (4/11) being the most frequently used. Notably, nine studies integrated clinical parameters to develop combined clinical–radiomics models (Supplementary Table S5).

### 3.5. Performance of Radiomics Models in Predicting Treatment Response

Our review included seven radiomics studies evaluating the efficacy of immunotherapy in HCC patients [17,18,19,20,22,26,27] (Table 3). Among these, one study focused on predicting treatment response to ICI monotherapy, assessed using the mRECIST criteria, and reported AUC values of 0.894 (95% CI: 0.797–0.991) and 0.883 (95% CI: 0.716–0.998) in the training and internal validation cohorts, respectively [17]. Three studies evaluated the performance of radiomics models for predicting treatment response to ICIs combined with molecular targeted therapy, assessed using the RECIST 1.1 criteria. AUC values ranged from 0.886 to 0.956 in training sets and from 0.792 to 0.802 in internal validation sets. Notably, a clinical–radiomics model integrating five clinical factors with imaging features demonstrated robust performance, achieving AUC values of 0.987 (95% CI: 0.968–1.000) and 0.884 (95% CI: 0.762–1.000) in the training and external validation cohorts, respectively [19]. In addition, three studies evaluated the utility of radiomics for predicting treatment response to ICIs combined with both molecular targeted therapy and locoregional therapy, assessed using the mRECIST criteria. Radiomics models achieved AUC values of 0.877–0.920 in training sets and 0.721–0.790 in internal validation sets. Clinical–radiomics models demonstrated superior performance, with AUC values of 0.950–0.960 in training sets and 0.840–0.850 in internal validation sets.

### 3.6. Performance of Radiomics Models in Predicting OS

Four studies evaluated the performance of radiomics models in predicting OS in HCC patients [18,21,23,24] (Table 4). In three studies focusing on ICIs combined with molecular targeted therapy, radiomics models achieved C-index values of 0.76–0.77 in training cohorts, 0.70 in internal validation cohorts, and 0.63–0.69 in external validation cohorts. Of note, incorporating clinical factors to develop clinical–radiomics models significantly enhanced predictive performance, with C-index values increasing to 0.78–0.82 in training cohorts, 0.82 in internal validation cohorts, and 0.67–0.74 in external validation cohorts. For OS prediction in patients receiving systemic therapy combined with locoregional therapy, the radiomics model yielded a C-index of 0.838 (95% CI: 0.806–0.870) in the training cohort and 0.817 (95% CI: 0.748–0.886) in the internal validation cohort. After integrating key clinical factors, including albumin–bilirubin (ALBI) grade and portal vein tumor thrombus (PVTT), model performance improved further, achieving C-index values of 0.867 (95% CI: 0.839–0.898) in the training cohort and 0.840 (95% CI: 0.782–0.897) in the validation cohort [21].

### 3.7. Performance of Radiomics Models in Predicting PFS

A total of four studies evaluated the performance of radiomics models in predicting PFS in HCC patients [23,24,25,26] (Table 5). Three studies focused on patients receiving ICIs combined with molecular targeted agents. In these studies, radiomics models achieved C-index values ranging from 0.67 to 0.837 in training sets, 0.64–0.830 in internal validation sets, and 0.54–0.66 in external validation sets. Clinical–radiomics combined models showed improved performance, with C-index values of 0.70–0.846 in training sets, 0.68–0.845 in internal validation sets, and 0.59–0.69 in external validation sets. Only one study developed a radiomics model to predict PFS in patients receiving systemic therapy combined with locoregional treatment. The initial radiomics model achieved a C-index of 0.59, which improved significantly to 0.75 after incorporating key clinical factors [26].

## 4. Discussion

Currently, no accurate and reliable biomarkers are available in clinical practice to guide individualized precision immunotherapy for HCC. Increasing evidence suggests that radiomics may provide valuable insights into tumor heterogeneity and help predict response and outcomes to immunotherapy [28,29,30]. This systematic review indicates that pretreatment CT/MRI-based radiomics models show overall promise for predicting immunotherapy outcomes in HCC, particularly for short-term responses, and model performance is generally improved when clinical variables are integrated. However, the evidence base remains constrained by substantial clinical and methodological heterogeneity, limited evaluation of long-term endpoints (OS/PFS), and a consistent gap between training and validation performance, highlighting concerns regarding generalizability. Quality appraisal using the RQS and METRICS further suggests that current studies have methodological limitations, with recurring shortcomings in external validation, prospective design, and transparency. Collectively, these findings support radiomics as a candidate imaging biomarker while underscoring the need for standardized workflows and geographically diverse, multicenter prospective validation prior to clinical adoption.

This systematic review synthesizes the available evidence on radiomics models based on pretreatment CT or MRI imaging for predicting immunotherapy efficacy in HCC. The 11 included studies demonstrated substantial heterogeneity in study design, particularly regarding the types of immunotherapy regimens and the metrics used to evaluate effectiveness. Currently, a variety of immunotherapy approaches are employed in clinical practice for HCC treatment [31]. The immunotherapy regimens in the included studies can be grouped into three categories: ICI monotherapy, ICIs combined with molecular targeted therapy, and ICIs combined with locoregional therapy. The metrics used to evaluate immunotherapy efficacy also varied across studies. Primary endpoints included OS, PFS, and treatment response assessed using the RECIST 1.1 or mRECIST criteria. While RECIST 1.1 and mRECIST focus on short-term responses, OS and PFS reflect long-term outcomes. Predictive biomarkers for long-term efficacy are particularly valuable, as they can help clinicians make informed treatment decisions [32]. However, there are relatively few studies evaluating time-to-event endpoints in HCC patients receiving immunotherapy monotherapy. It is also important to note that immunotherapy can produce delayed responses or pseudoprogression, meaning that traditional solid tumor evaluation criteria like RECIST may not fully capture therapeutic effects. The recently proposed iRECIST criteria were specifically designed to evaluate immunotherapy responses, but none of the included studies applied this framework.

### 4.1. Study Heterogeneity and Predictive Performance

Owing to substantial clinical and methodological heterogeneity among the included studies, a pooled analysis of effect sizes was not conducted. Only one eligible study evaluated radiomics for predicting outcomes under ICI monotherapy [17], and therefore, these findings should be considered hypothesis-generating and not generalizable. This limitation likely reflects current clinical practice in hepatocellular carcinoma, where the limited efficacy of single-agent ICIs has led to a shift toward combination regimens [3]. In studies predicting immunotherapy response, models for HCC patients receiving ICIs combined with molecular targeted therapy demonstrated excellent performance in training cohorts, with AUC values ranging from 0.880 to 0.956. For patients treated with ICIs combined with both molecular targeted therapy and locoregional therapy, models achieved AUC values of 0.877–0.920. Corresponding validation sets showed slightly lower AUC values of 0.792–0.820 and 0.721–0.790, respectively. These findings suggest that the models may exhibit some degree of overfitting, although they generally maintain good discriminative performance. The predictive performance of models for patients receiving combined locoregional therapy was relatively lower, which may reflect the increased difficulty in assessing efficacy due to the complexity of combination regimens. Regarding long-term efficacy prediction, studies remain limited, likely due to the need for extended follow-up and higher research costs. In training cohorts of HCC patients treated with ICIs combined with targeted therapy, radiomics models predicting OS and PFS achieved C-index values of 0.76–0.838 and 0.59–0.837, respectively, while validation cohorts yielded C-index values of 0.63–0.817 and 0.54–0.830.

A horizontal comparison indicated that predictive performance for long-term efficacy indicators was generally lower than that for short-term outcomes, reflecting the inherent challenges of long-term prognosis prediction. Despite variability in immunotherapy regimens and efficacy evaluation metrics across studies, the overall findings support the good discriminative ability of radiomics models in predicting HCC immunotherapy efficacy, suggesting their potential clinical utility. Furthermore, integrating clinical factors consistently improved model performance, highlighting the added value of combining clinical information with radiomic features.

### 4.2. Methodological Quality Assessment (RQS and METRICS)

Next, we assessed the methodological quality of the included studies using two tools: the RQS and METRICS. The RQS assessment revealed a median score of 15, corresponding to 41.7%, with only one study scoring above 50%. These results indicate that the overall quality of current radiomics models remains suboptimal, with notable deficiencies in areas such as phantom studies, multi-timepoint imaging, prospective study design, cost-effectiveness analysis, biological relevance, and open data sharing. Our findings are consistent with previous systematic reviews evaluating radiomics models for immunotherapy response in lung cancer (median RQS: 11) [28], histopathological grading in HCC (median RQS: 10) [33], and lymph node metastasis in colorectal cancer (median RQS: 18) [34]. To enhance methodological rigor and facilitate clinical translation, future radiomics studies should ensure adherence to several core domains. Specifically, investigators should (1) provide sufficiently detailed reporting of image acquisition and reconstruction protocols to support reproducibility; (2) implement rigorous validation strategies, at least including internal validation and, whenever feasible, independent external validation to establish generalizability; (3) minimize overfitting and data leakage through strict separation between training and validation phases, with feature selection and hyperparameter tuning confined exclusively to the training set; (4) evaluate the reproducibility and robustness of segmentation and radiomic features using quantitative metrics (e.g., intraclass correlation coefficients and/or test–retest or multi-timepoint assessments); and (5) report model performance comprehensively, including both discrimination and calibration, along with transparent disclosure of key methodological parameters.

In contrast to the more established RQS, the METRICS is a relatively novel assessment tool. Evaluation using the METRICS yielded a median score of 72.5%, indicating higher methodological quality compared to the RQS and reflecting its distinct scoring framework for assessing study rigor. As the METRICS is a relatively new tool, its application in radiomics research is still in the early stages.

### 4.3. Major Limitations of Current Evidence

Through systematic analysis of low-scoring items in both quality assessment tools, we identified the following major limitations in current radiomics research: (1) Heterogeneity in imaging data: None of the included studies adhered to standardized image acquisition protocols, and several studies used scanners from different manufacturers, introducing variability in the raw imaging data. This heterogeneity largely reflects the inherent limitations of retrospective study designs in data selection. Furthermore, no study has explicitly examined how this variability affects radiomic features or subsequent analytical results. (2) Insufficient model generalizability: Most radiomics models were developed using small, highly homogeneous datasets and lacked external validation across multiple centers or diverse populations. Among the eleven included studies, only four performed external validation. Although some studies incorporated multicenter data, limited sample sizes often necessitated pooling the data for model training, making it difficult to evaluate the models’ generalizability in real-world clinical settings. (3) Lack of biological interpretability: Establishing correlations between radiomic features and underlying biological mechanisms would link model decisions to pathophysiological processes, enhancing clinical trust and translational potential. However, only one study examined the biological relevance of radiomic features. (4) Limited clinical usability: Most studies relied on manual or semi-automated image segmentation, which is time-consuming, labor-intensive, and highly dependent on operator expertise, limiting reproducibility and efficiency in routine clinical practice. Furthermore, physiological and technical factors, such as respiratory motion, gastrointestinal peristalsis, and patient positioning, can cause significant liver deformation and signal fluctuations over short periods, resulting in the instability of radiomic features extracted from single scans. No study employed multi-timepoint imaging to ensure feature robustness. While most studies used multiphase images to construct radiomics models, current designs tend to be overly complex. Although this complexity may improve performance, it increases computational burden and costs, with no comparisons to simpler models or cost-effectiveness analysis. (5) Inadequate transparency: The radiomics modeling process is inherently complex, and open access to data and methods is essential for validation, methodological refinement, and clinical translation. Unfortunately, most studies did not provide easily accessible open-source data.

### 4.4. Clinical Implications and Future Directions

Immunotherapy is a cornerstone of systemic treatment for HCC. Radiomics-based risk stratification could complement conventional clinical variables and biomarker profiles to guide patient selection, treatment escalation or de-escalation, and clinical trial enrichment. If prospectively validated, such models may reduce avoidable treatment-related toxicity and unnecessary financial costs, including drug expenditures and the management of immune-related adverse events, while helping clinicians avoid delays that could cause patients to miss the optimal therapeutic window by promptly identifying individuals most likely to benefit from a given immunotherapy strategy. To accelerate clinical translation, future studies should (1) adopt rigorous designs through preregistered, prospective, multicenter protocols with prespecified endpoints and locked analysis plans to minimize selection bias and analytic flexibility; (2) standardize and quantify imaging variability by harmonizing acquisition parameters and, where feasible, incorporate phantom-based or test–retest scans to characterize scanner-specific effects and inform protocol harmonization; (3) implement multi-timepoint imaging protocols aligned with immunotherapy dynamics, including at minimum a baseline scan within 2 weeks before treatment initiation, an early on-treatment scan coinciding with the first response assessment, and follow-up scans every 8–12 weeks, with an additional confirmation scan 4–8 weeks after immune-unconfirmed progressive disease (iUPD) when immune-adapted criteria are used, enabling delta-radiomics and longitudinal modeling while allowing for the explicit evaluation of feature stability under motion-related liver deformation; (4) improve usability and efficiency by implementing externally validated fully automated or strictly standardized semi-automated segmentation workflows and reporting segmentation reliability when manual intervention is required; and (5) demonstrate clinical and economic value by supplementing the AUC with decision curve analysis and conducting model-based cost–utility analyses that compare radiomics-guided treatment allocation to standard-of-care pathways, including treatment costs, adverse-event management, downstream imaging, and quality-adjusted life years (QALYs), while transparently sharing de-identified data, segmentations, and code in accordance with FAIR principles to support independent verification and benchmarking.

### 4.5. Limitations of This Review

This systematic review has several limitations that warrant consideration. First, significant clinical heterogeneity existed among the included studies due to variations in treatment regimens and evaluation criteria. In the current therapeutic landscape of HCC, multiple immunotherapy strategies are used alongside diverse efficacy assessment metrics. Since radiomics-based prediction of immunotherapy response in HCC is still in its early stages, imposing strict restrictions on specific treatment protocols or outcome measures would have reduced the number of eligible studies to fewer than five. Therefore, during study selection, we did not strictly limit the types of immunotherapy regimens or efficacy endpoints; instead, we conducted stratified analyses according to different treatment–outcome combinations. Second, the evidence is geographically concentrated, with most studies conducted in China. This may reflect the high burden of HCC and early, focused investment in radiomics research in this region. Geographic clustering may limit the external validity of the findings, as regional differences in patient demographics, etiology, clinical pathways, and imaging protocols can affect model performance and transportability. Therefore, although the findings are informative given the current evidence base, their generalizability to other regions requires validation through prospective, multicenter studies across diverse populations and clinical settings with harmonized imaging workflows. Finally, this review did not include prediction models based on ultrasound or PET imaging. Ultrasound image quality is highly operator-dependent, and PET scanning is costly and not routinely performed in all HCC patients. In contrast, CT and MRI are integral to the HCC diagnostic and therapeutic workflow. Accordingly, we focused exclusively on radiomics models constructed from CT/MRI images to enhance the generalizability of our findings.

## 5. Conclusions

Radiomics signatures derived from pretreatment CT or MRI are promising candidate imaging biomarkers for predicting immunotherapy response in hepatocellular carcinoma. However, clinical translation requires geographically diverse, multicenter prospective validation with rigorous external testing across institutions and standardized radiomics pipelines to ensure reproducibility and transportability.

## Figures and Tables

**Table 1:** Overall characteristics of included studies.

First Author	Publication Year	Country	Study Design	Study Center	Treatment	No. of Patients	Gender (Male/Female)	Age	Predicted Outcome
Yuan, G.S. [17]	2021	China	Retrospective	S	ICIs	58	52/6	55; 52 ^#^	mRECIST
Xu, B. [19]	2022	China	Retrospective	M	ICIs + Targeted therapy	170	154/16	55; 57 ^#^	RECIST1.1, OS, PFS
Dong, W. [18]	2023	China	Retrospective	S	ICIs + Targeted therapy	55	50/5	53	RECIST1.1, OS
Liao, N.Q. [20]	2024	China	Retrospective	S	ICIs + Targeted therapy	120	99/21	48; 47 ^#^	RECIST1.1
Vithayathil, M. [23]	2025	UK	Retrospective	M	ICIs + Targeted therapy	152	130/22	67; 63 ^#^	OS, PFS
Xu, X.N. [25]	2025	China	Retrospective	S	ICIs + Targeted therapy	111	105/6	56	PFS
Xu, J. [24]	2025	China	Retrospective	M	ICIs + Targeted therapy	859	736/123	58; 57 ^#^	OS, PFS
Lu, D.Y. [22]	2025	China	Retrospective	M	ICIs + Targeted therapy + Locoregional therapy	115	104/11	56	mRECIST
Yin, L.N. [26]	2025	China	Retrospective	M	ICIs + Targeted therapy + Locoregional therapy	122	104/18	54	mRECIS, PFS
Zhu, Y.M. [27]	2025	China	Retrospective	M	ICIs + Targeted therapy + Locoregional therapy	102	92/10	53; 57 ^#^	mRECIST
Ding, G.Y. [21]	2025	China	Retrospective	S	ICIs + Targeted therapy + Locoregional therapy	150	133/17	>60; 38%	OS

S, single; M, multiple; ICIs, immune checkpoint inhibitors; #, training cohort and validation cohort, respectively; mRECIST, Modified Response Evaluation Criteria In Solid Tumors; RECIST1.1, Modified Response Evaluation Criteria In Solid Tumors Version1.1; OS, overall survival; PFS, progression-free survival.

**Table 2:** The characteristics of the radiomics model pipeline.

Study ID	Imaging Modality	Imaging Sequence	Segmentation Method	Feature Extraction	Feature Extracted	Feature Selection	Feature in the Model	Modeling Algorithms
Yuan et al. [17]	CT	NC, AP	Manually	Pyradiomics	3160	ICC, Spearman correlation test, *t* test, and LASSO	9	LASSO, RF, SVM, and DT
Xu et al. [19]	MRI	AP, DP	Manually	Pyradiomics	2236	ICC, *t* test, and LASSO	17	Neural network model
Dong et al. [18]	CT	AP, PVP	Semi-auto	Pyradiomics	2458	ICC and LASSO	10	SVM, NB, Rpart, Ctree, RF, KNN, neuralnet, boosting, bagging, and logistics
Liao et al. [20]	CT	AP, PVP, DP	Unclear	ResNet-18	-	-	-	ResNet-18, VGG19, ResNet-50, and Mobilenetv3
Vithayathil, M. et al. [23]	CT	PVP	semi-auto	TexLAB	892	LASSO, elastic net, RFE, PCA, Boruta, mutual information, Pearson and Spearman correlations, Kendall correlation, ANOVA F-test, variance threshold, and forward selection	-	XGBoost, logistic regression, Naïve bayes, neural network, random forest, Ridge regression, SVM, and Kmeans clustering
Xu et al. [25]	MRI	AP, PVP, DP	Manually	Pyradiomics	2736	Univariable Cox model and LASSO	32	RSF and Cox regression
Xu et al. [24]	CT	AP, PVP, DP	Semi-auto	Pyradiomics	642	Univariate Cox model, VIF, and RSF	16	EfficientNet B1 Model, semi-supervised hybrid model, CNN-Transformer Model, and RSF
Lu et al. [22]	MRI	T1WI, T2WI, DWI	Manually	3D slicer	851	ICC, *t* test, LASSO, and RFE	12	SVM, KNN, XGBoost, and RF
Yin et al. [26]	CT	AP, PVP, DP	Manually	ResNet50	-	MLP and Cox regression	6	Cox regression
Zhu et al. [27]	MRI	T1WI, AP, PVP, DP	Manually	Pyradiomics	428	ICC and LASSO	5	Extra Trees, Crossformer, and ResNet50
Ding et al. [21]	CT	AP, PVP	Manually	Pyradiomics	3376	ICC, Pearson correlation, univariate Cox analysis, LASSO Cox regression	5	LASSO Cox regression

CT, computed tomography; MRI, magnetic resonance imaging; NC, non-contrast phase; AP, arterial phase; DP, delayed phase; PVP, portal venous phase; ICC, intraclass correlation coefficient; LASSO, least absolute shrinkage and selection operator; RFE, recursive feature elimination; PCA, principal component analysis; RF, random forest; SVM, support vector machines; DT, decision tree; VIF, variance Inflation Factor; KNN, K-Nearest Neighbors.

**Table 3:** Performance of radiomics models in predicting treatment response.

Study ID	Radiomics			Clinical–Radiomics			Calibration Curve	Decision Curve Analysis	Model Form
	AUC (Training)	AUC (Internal Validation)	AUC (External Validation)	AUC (Training)	AUC (Internal Validation)	AUC (External Validation)			
**ICIs**									
Yuan, G.S. et al. [17]	0.772	0.705	-	0.894 [0.797, 0.991]	0.883 [0.716, 0.998]	-	Yes	Yes	Nomogram
**ICIs combined with molecular targeted therapy**									
Xu, B. et al. [19]	0.886 [0.815, 0.957]	-	0.820 [0.648, 0.984]	0.987 [0.968, 1.000]	-	0.884 [0.762, 1.000]	Yes	Yes	-
Dong, W. et al. [18]	0.933	0.792	-	-	-	-	No	No	-
Liao, N.Q. et al. [20]	0.956 [0.931, 0.981]	0.802 [0.753, 0.851]	-	-	-	-	No	No	-
**ICIs combined with molecular targeted therapy and local therapy**									
Lu, D.Y. et al. [22]	0.92 [0.86, 0.97]	0.79 [0.61, 0.95]	-	0.95 [0.68, 0.98]	0.84 [0.91, 0.99]	-	No	No	-
Yin, L.N. et al. [26]	-	-	-	0.96	0.87	0.85	No	No	-
Zhu, Y.M. et al. [27]	0.877 [0.795, 0.958]	0.721 [0.556, 0.886]	-	-	-	-	Yes	Yes	-

ICIs, immune checkpoint inhibitors; AUC, area under the curve.

**Table 4:** Performance of radiomics models in predicting OS.

Study ID	Radiomics			Clinical–Radiomics			Calibration Curve	Decision Curve Analysis	Model Form
	C-Index (Training)	C-Index (Internal Validation)	C-Index (External Validation)	C-Index (Training)	C-Index (Internal Validation)	C-Index (External Validation)			
**ICIs combined with molecular t** **argete** **d therapy**									
Dong, W. et al. [18]	-	-	-	0.81	-	-	No	Yes	-
Vithayathil, M. et al. [23]	0.77 [0.69, 0.84]	-	0.63 [0.55, 0.70]	0.78 [0.67–0.85]	-	0.67 [0.60, 0.74]	Yes	Yes	-
Xu, J. et al. [24]	0.76 [0.73, 0.79]	0.70 [0.64, 0.76]	0.69 [0.64, 0.73]	0.82 [0.79, 0.84]	0.73 [0.68, 0.79]	0.74 [0.70, 0.78]	No	No	Formula
**ICIs combined with molecular targeted therapy and local therapy**									
Ding, G.Y. et al. [21]	0.838 [0.806, 0.870]	0.817 [0.748, 0.886]	-	0.867 [0.839, 0.898]	0.840 [0.782, 0.897]	-	Yes	Yes	Nomogram

ICIs, immune checkpoint inhibitors; C-index, concordance index.

**Table 5:** Performance of radiomics models in predicting PFS.

Study ID	Radiomics			Clinical–Radiomics			Calibration Curve	Decision Curve Analysis	Model Form
	C-Index (Training)	C-Index (Internal Validation)	C-Index (External Validation)	C-Index (Training)	C-Index (Internal Validation)	C-Index (External Validation)			
**ICIs combined with molecular targeted therapy**									
Vithayathil, M. et al. [23]	0.67 [0.58, 0.76]	-	0.54 [0.48, 0.62]	0.70 [0.62, 0.78]	-	0.59 [0.51, 0.67]	Yes	Yes	-
Xu, X.N. et al. [25]	0.837	0.830	-	0.846 [0.804,0.879]	0.845 [0.767, 0.893]	-	Yes	Yes	Nomogram
Xu, J. et al. [24]	0.69 [0.66, 0.71]	0.64 [0.58, 0.69]	0.66 [0.61, 0.70]	0.72 [0.69, 0.74]	0.68 [0.62, 0.74]	0.69 [0.65, 0.73]	No	No	Formula
**ICIs combined with molecular targeted therapy and local therapy**									
Yin, L.N. et al. [26]	0.59	-	-	0.75	-	-	Yes	No	Nomogram

ICIs, immune checkpoint inhibitors; C-index, concordance index.

## Data Availability

No new data were created or analyzed in this study. Data sharing is not applicable to this article as it is a review paper based on previously published literature.

## Supplementary Materials

The following supporting information can be downloaded at: https://www.mdpi.com/article/10.3390/cancers18020186/s1, Supplementary Table S1. Literature search strategy. Supplementary Table S2. Final scoring of specific items in the Radiomics Quality Score (RQS). Supplementary Table S3. Final scoring of specific items in the METhodological RadiomICs Score (METRICS). Supplementary Table S4. Types of imaging acquisition devices in included studies. Supplementary Table S5. Clinical factors integrated into clinical-radiomics models.
