# Supplementary material for: Radiomics for Predicting the Efficacy of Immunotherapy in Hepatocellular Carcinoma: A Systematic Review and Radiomics Quality Score Assessment
Source: Cancers (Basel). 2026 Jan 6;18(2):186. doi: 10.3390/cancers18020186 (PMC12839198; doi:10.3390/cancers18020186)
Supplement: Supplementary file 1 [file cancers-18-00186-s001.zip › cancers-4072108-supplementary.pdf]

# Supplementary Materials

## Supplementary Information for: Radiomics for Predicting the Efficacy of Immunotherapy in Hepatocellular Carcinoma: A Systematic Review and Radiomics Quality Score Assessment

### List of Supplementary Materials

**Supplementary Table S1**. Literature search strategy.
**Supplementary Table S2**. Final scoring of specific items in the Radiomics Quality Score (RQS).
**Supplementary Table S3**. Final scoring of specific items in the METhodological RadiomICs Score (METRICS).
**Supplementary Table S4**. Types of imaging acquisition devices in included studies.
**Supplementary Table S5**. Clinical factors integrated into clinical-radiomics models.

**Supplementary Table S1**. Literature search strategy.

| **Database** | |
|---|---|
| **Pubmed** | 1.“Radiomics”[MeSH]<br>2.(Radiomic* [tw]) OR (Artificial intelligence [tw]) OR (deep learning [tw]) OR (convolutional neural network [tw]) OR (machine learning [tw]) OR (automatic detection [tw])<br>3.#1 OR #2<br>4.(Magnetic Resonance Imaging [MeSH]) OR (Tomography, X-Ray Computed [MeSH])<br>5.(Magnetic Resonance Imaging [tw]) OR (Tomography, X-Ray Computed [tw]) OR (CT [tw]) OR (MRI [tw]) OR (Computed tomography [tw])<br>6.#4 OR #5<br>7.“carcinoma, hepatocellular” [MeSH]<br>8.(Carcinoma*, Hepatocellular [tw]) OR (Hepatocellular Carcinoma* [tw]) OR (Hepatoma* [tw]) OR (Liver Cell Carcinoma* [tw]) OR (Carcinoma*, Liver Cell [tw]) OR (Cell Carcinoma*, Liver [tw])<br>9.#7 OR #8<br>10.“Immune Checkpoint Inhibitors”[MeSH terms]<br>11.(Checkpoint Inhibitor*, Immune [tw]) OR (Immune Checkpoint Blocker* [tw]) OR (Checkpoint Blocker*, Immune [tw]) OR (Immune Checkpoint Inhibitor* [tw]) OR (Immune Checkpoint Blockade [tw]) OR (Checkpoint Blockade, Immune [tw]) OR (Immune Checkpoint Inhibition [tw]) OR (Checkpoint Inhibition, Immune [tw]) OR (PD-1 Inhibitor* [tw]) OR (PD 1 Inhibitor* [tw]) OR (Programmed Cell Death Protein 1 Inhibitor* [tw]) OR (Programmed Death-Ligand 1 Inhibitor* [tw]) OR (Inhibitor*, PD-1 [tw]) OR (PD-L1 Inhibitor* [tw]) OR (PD L1 Inhibitor* [tw]) OR (PD-1-PD-L1 Blockade [tw]) OR (Blockade, PD-1-PD-L1 [tw]) OR (PD-1 [tw]) OR (PD-L1 [tw]) OR (CTLA-4 [tw]) OR (Nivolumab [tw]) OR (Pembrolizumab [tw]) OR (Sintilimab [tw]) OR (Camrelizumab [tw]) OR (Tislelizumab [tw]) OR (Toripalimab [tw]) OR (Penpulimab [tw]) OR (Atezolizumab [tw]) OR (Durvalumab [tw]) OR (Envafolimab [tw]) OR (Ipilimumab [tw]) OR (Tremelimumab [tw])<br>12.#10 OR #11<br>13.#3 AND #6 AND #9 AND #12 |
| **Embase** | 1.'radiomics'/exp<br>2.'Radiomic*':ab,ti OR 'Artificial intelligence':ab,ti OR 'deep learning':ab,ti OR 'convolutional neural network':ab,ti OR 'machine learning':ab,ti OR 'automatic detection':ab,ti<br>3.#1 OR #2<br>4.'liver cell carcinoma'/exp<br>5.'liver cell carcinoma*':ab,ti OR 'carcinoma*, hepatocellular':ab,ti OR 'Hepatocellular Carcinoma*':ab,ti OR 'Hepatoma*':ab,ti OR 'Carcinoma*, Liver Cell':ab,ti OR 'Cell Carcinoma*, Liver':ab,ti<br>6.#4 OR #5<br>7.'nuclear magnetic resonance imaging'/exp OR 'computer assisted tomography'/exp<br>8.'nuclear magnetic resonance imaging':ab,ti OR 'imaging, magnetization transfer':ab,ti OR 'magnetic resonance imaging':ab,ti OR 'magnetic resonance tomography':ab,ti OR 'magnetization transfer imaging':ab,ti OR 'mr imaging':ab,ti OR 'MRI':ab,ti OR 'NMR imaging':ab,ti OR 'computer assisted tomography':ab,ti OR 'CAT scan':ab,ti OR 'CAT scanning':ab,ti OR 'computed axial tomography':ab,ti OR 'computed tomographic scan':ab,ti OR 'computed tomography':ab,ti OR 'computed tomography |

| | |
|---|---|
| | scan':ab,ti OR 'computer tomography':ab,ti OR 'computerised axial tomography':ab,ti OR 'computerised tomography':ab,ti OR 'computerized axial tomography':ab,ti OR 'computerized tomography':ab,ti OR 'computerized tomography scan':ab,ti OR 'CT':ab,ti<br>9.#7 OR #8<br>10. 'immune checkpoint inhibitor'/exp<br>11.'immune checkpoint blocker':ab,ti OR 'immune checkpoint inhibitors':ab,ti OR 'immune checkpoint inhibitor':ab,ti OR 'PD-1 Inhibitor*':ab,ti OR 'PD 1 Inhibitor*':ab,ti OR 'Programmed Cell Death Protein 1 Inhibitor*':ab,ti OR 'Programmed Death-Ligand 1 Inhibitor*':ab,ti OR 'Inhibitor, PD-1':ab,ti OR 'PD-1-PD-L1 Blockade':ab,ti OR 'Blockade, PD-1-PD-L1':ab,ti OR 'PD-1':ab,ti OR 'PD-L1':ab,ti OR 'CTLA-4':ab,ti OR 'Nivolumab':ab,ti OR 'Pembrolizumab':ab,ti OR 'Sintilimab':ab,ti OR 'Camrelizumab':ab,ti OR 'Tislelizumab':ab,ti OR 'Toripalimab':ab,ti OR 'Penpulimab':ab,ti OR 'Atezolizumab':ab,ti OR 'Durvalumab':ab,ti OR 'Envafolimab':ab,ti OR 'Ipilimumab':ab,ti OR 'Tremelimumab':ab,ti<br>12.#10 OR #11<br>13.#3 AND #6 AND #9 AND #12 |
| **Web of Science Core Collection** | 1.Topic= (Artificial intelligence OR deep learning OR convolutional neural network OR machine learning OR automatic detection OR radiomic*)<br>2.Topic= (Hepatocellular Carcinoma* OR (Carcinoma*, Hepatocellular) OR Hepatoma* OR Liver Cell Carcinoma* OR (Carcinoma*, Liver Cell) OR (Cell Carcinoma*, Liver))<br>3.Topic= (Magnetic Resonance Imaging OR (Tomography, X-Ray Computed) OR CT OR MRI OR Computed tomography)<br>4.Topic= ( Immune Checkpoint Inhibitor* OR (Checkpoint Inhibitor*, Immune* ) OR Immune Checkpoint Blocker* OR ( Checkpoint Blocker*, Immune ) OR Immune Checkpoint Blockade OR ( Checkpoint Blockade, Immune ) OR Immune Checkpoint Inhibition OR ( Checkpoint Inhibition, Immune ) OR PD-1 Inhibitor* OR PD 1 Inhibitor* OR Programmed Cell Death Protein 1 Inhibitor* OR Programmed Death-Ligand 1 Inhibitor* OR ( Inhibitor*, PD-1 ) OR PD-L1 Inhibitor* OR PD L1 Inhibitor* OR PD-1-PD-L1 Blockade OR ( Blockade, PD-1-PD-L1 ) OR PD-1 OR PD-L1 OR CTLA-4 OR Nivolumab OR Pembrolizumab OR Sintilimab OR Camrelizumab OR Tislelizumab OR Toripalimab OR Penpulimab OR Atezolizumab OR Durvalumab OR Envafolimab OR Ipilimumab OR Tremelimumab)<br>5.#1 AND #2 AND #3 AND #4 |
| **Cochrane CENTRAL** | 1.MeSH descriptor: [Radiomics] explode all trees<br>2.((Radiomic*) OR ( Artificial intelligence ) OR ( deep learning ) OR ( convolutional neural network ) OR ( machine learning ) OR ( automatic detection )):ti,ab,kw<br>3.#1 OR #2<br>4.MeSH descriptor: [Carcinoma, Hepatocellular] explode all trees<br>5.((Carcinomas, Hepatocellular) OR (Hepatocellular Carcinomas) OR (Hepatocellular Carcinoma) OR (Hepatoma) OR (Hepatomas) OR (Liver Cell Carcinoma) OR (Carcinoma, Liver Cell) OR (Carcinomas, Liver Cell) OR (Cell Carcinoma, Liver) OR (Cell Carcinomas, Liver) OR (Liver Cell Carcinomas)):ti,ab,kw<br>6.#3 OR #4<br>7.MeSH descriptor: [Magnetic Resonance Imaging] explode all trees<br>8.((Magnetic Resonance Imaging) OR (MRI)):ti,ab,kw |

| | |
|---|---|
| | 9.MeSH descriptor: [Tomography, X-Ray Computed] explode all trees<br>10.((Tomography, X-Ray Computed) OR (CT) OR (Computed tomography)):ti,ab,kw<br>11.#7 OR #8 OR #9 OR #10<br>12.MeSH descriptor: [Immune Checkpoint Inhibitors] explode all trees<br>13.(Checkpoint Inhibitor*, Immune) OR (Immune Checkpoint Blocker*) OR (Checkpoint Blocker*, Immune) OR (Immune Checkpoint Inhibitor*) OR (PD-1 Inhibitor*) OR (PD 1 Inhibitor*) OR (Programmed Cell Death Protein 1 Inhibitor*) OR (Programmed Cell Death Protein 1 Inhibitor*) OR (Inhibitor*, PD-1) OR (Immune Checkpoint Blockade) OR (Checkpoint Blockade, Immune) OR (Immune Checkpoint Inhibition)OR (Checkpoint Inhibition, Immune) OR (PD-L1 Inhibitor*) OR (PD L1 Inhibitor*)OR (Programmed Death-Ligand 1 Inhibitor*) OR (Programmed Death Ligand 1 Inhibitor*) OR (PD-1 Blockade) OR (PD-L1 Blockade) OR (Blockade, PD-1) OR (Blockade, PD-L1) (PD-1) OR (PD-L1) OR (CTLA-4) OR (Nivolumab) OR (Pembrolizumab) OR (Sintilimab) OR (Camrelizumab) OR (Tislelizumab) OR (Toripalimab) OR (Penpulimab) OR (Atezolizumab) OR (Durvalumab) OR (Envafolimab) OR (Ipilimumab) OR (Tremelimumab)<br>14.#12 AND #13<br>15.#3 AND #6 AND #11 AND #14 |

**Supplementary Table S2**. Final scoring of specific items in the Radiomics Quality Score (RQS).

| Study | Item 1 | Item2 | Item3 | Item4 | Item5 | Item6 | Item7 | Item8 | Item9 | Item10 | Item11 | Item12 | Item13 | Item14 | Item15 | Item16 | overall |
|---|---|---|---|---|---|---|---|---|---|---|---|---|---|---|---|---|---|
| Yuan GS | 1 | 1 | 0 | 0 | 3 | 1 | 0 | 1 | 1 | 2 | 0 | 2 | 2 | 2 | 0 | 0 | 16 |
| Dong W | 1 | 1 | 0 | 0 | 3 | 1 | 0 | 0 | 1 | 0 | 0 | 2 | 2 | 0 | 0 | 0 | 11 |
| Liao NQ | 1 | 1 | 0 | 0 | 3 | 0 | 0 | 0 | 2 | 0 | 0 | 2 | 2 | 0 | 0 | 0 | 11 |
| Xu B | 1 | 1 | 0 | 0 | 3 | 1 | 0 | 1 | 2 | 1 | 0 | 5 | 2 | 2 | 0 | 0 | 19 |
| Vithayathil M | 1 | 1 | 0 | 0 | 3 | 1 | 0 | 1 | 2 | 1 | 0 | 3 | 2 | 2 | 0 | 0 | 17 |
| Xu XN | 1 | 1 | 0 | 0 | 3 | 1 | 0 | 1 | 1 | 2 | 0 | 2 | 2 | 2 | 0 | 1 | 17 |
| Xu J | 1 | 1 | 0 | 0 | 3 | 1 | 1 | 1 | 1 | 0 | 0 | 5 | 2 | 0 | 0 | 1 | 17 |
| Lu DY | 1 | 1 | 0 | 0 | 3 | 1 | 0 | 0 | 2 | 0 | 0 | 3 | 2 | 0 | 0 | 0 | 13 |
| Yin LN | 1 | 1 | 0 | 0 | 3 | 1 | 0 | 0 | 1 | 1 | 0 | 3 | 2 | 0 | 0 | 0 | 13 |
| Zhu YM | 1 | 1 | 0 | 0 | 3 | 1 | 0 | 0 | 1 | 1 | 0 | 3 | 2 | 2 | 0 | 0 | 15 |
| Ding GY | 1 | 1 | 0 | 0 | 3 | 1 | 0 | 1 | 1 | 1 | 0 | 2 | 2 | 2 | 0 | 0 | 15 |

**Supplementary Table S3**. Final scoring of specific items in the METhodological RadiomICs Score (METRICS).

| | **Yuan GS** | **Dong W** | **Liao NQ** | **Xu B** | **Vithayathil M** | **Xu XN** | **Xu J** | **Lu DY** | **Yin LN** | **Zhu YM** | **Ding GY** |
|---|---|---|---|---|---|---|---|---|---|---|---|
| | | | | | Study Design | | | | | | |
| Item#1 | yes | no | no | no | no | no | no | no | no | no | no |
| Item#2 | yes | yes | yes | yes | yes | yes | yes | yes | yes | yes | yes |
| Item#3 | yes | yes | yes | yes | yes | yes | yes | yes | yes | yes | yes |
| | | | | | Imaging Data | | | | | | |
| Item#4 | no | no | no | yes | yes | no | yes | yes | yes | yes | no |
| Item#5 | no | no | no | no | no | no | no | no | no | no | no |
| Item#6 | yes | no | yes | yes | no | yes | yes | yes | no | yes | yes |
| Item#7 | yes | yes | yes | yes | yes | yes | yes | yes | yes | yes | yes |
| | | | | | SegmentationC | | | | | | |
| Condition #1 | yes | yes | no | yes | yes | yes | yes | yes | yes | yes | yes |
| Condition #2 | no | no | no | no | no | no | no | no | no | no | no |
| Item#8 | yes | yes | n/a | yes | yes | yes | yes | yes | yes | yes | yes |
| Item#9 | n/a | n/a | n/a | n/a | n/a | n/a | n/a | n/a | n/a | n/a | n/a |
| Item#10 | no | no | n/a | no | no | no | yes | no | no | no | no |
| | | | | | Image Processing and Feature Extraction | | | | | | |
| Condition #3 | yes | yes | no | yes | yes | yes | yes | yes | no | yes | yes |
| Item#11 | yes | yes | yes | yes | yes | yes | yes | yes | no | yes | yes |
| Item#12 | yes | yes | n/a | yes | yes | yes | yes | yes | n/a | yes | yes |
| Item#13 | no | no | no | yes | yes | no | yes | no | no | no | no |
| | | | | | Feature Processing | | | | | | |
| Condition #4 | yes | yes | no | yes | yes | yes | yes | yes | no | yes | yes |
| Condition #5 | no | no | yes | no | no | no | no | no | yes | no | no |
| Item#14 | yes | yes | n/a | yes | no | no | no | yes | n/a | yes | yes |
| Item#15 | yes | yes | n/a | yes | yes | yes | yes | yes | n/a | yes | yes |
| Item#16 | yes | yes | n/a | yes | yes | yes | yes | yes | n/a | yes | yes |
| Item#17 | n/a | n/a | yes | n/a | n/a | n/a | n/a | n/a | no | n/a | n/a |
| | | | | | Preparation for Modeling | | | | | | |
| Item#18 | yes | yes | yes | yes | yes | yes | yes | yes | yes | yes | yes |
| Item#19 | yes | No | yes | No | No | No | yes | yes | yes | yes | yes |
| | | | | | Metrics and Comparison | | | | | | |
| Item#20 | yes | yes | yes | yes | yes | yes | yes | yes | yes | yes | yes |
| Item#21 | yes | no | yes | yes | yes | yes | yes | yes | no | yes | yes |
| Item#22 | yes | no | no | yes | yes | yes | no | no | yes | yes | yes |
| Item#23 | no | yes | yes | no | no | no | no | no | no | no | no |

| | | | | | | | | | | | |
|---|---|---|---|---|---|---|---|---|---|---|---|
| Item#24 | yes | no | no | yes | yes | yes | yes | yes | yes | no | yes |
| Item#25 | no | no | no | no | no | no | no | no | yes | no | no |
| Testing | | | | | | | | | | | |
| Item#26 | yes | yes | yes | no | no | yes | yes | yes | yes | no | yes |
| Item#27 | no | no | no | yes | yes | no | no | no | yes | yes | no |
| Open Science | | | | | | | | | | | |
| Item#28 | no | no | no | no | no | no | no | no | no | no | no |
| Item#29 | no | no | no | no | no | yes | yes | no | no | no | no |
| Item#30 | no | no | no | no | no | no | no | no | no | no | no |
| Total METRICS score | 73.6% | 56.0% | 62.3% | 79.5% | 72.8% | 65.4% | 76.7% | 72.5% | 65.5% | 75.2% | 69.8% |
| Quality category | Good | Moderate | Good | Excellent | Good | Good | Good | Good | Good | Good | Good |

**Supplementary Table S4**. Types of imaging acquisition devices in included studies.

| Study ID | No. of device | Device name |
|---|---|---|
| Yuan GS et al 2021 | 1 | 64-multidetector CT imaging system (SOMATOM Definition, SIEMENS) |
| Xu B et al 2022 | 3 | 3.0-T scanner (Verio, Siemens Medical Solutions, Erlangen, Germany or uMR770, United Imaging Healthcare, Shanghai, China) or a 1.5-T scanner (Magnetom Aera, Siemens Medical Solutions, Erlangen, Germany) |
| Dong W et al 2023 | 1 | 64-multidetector CT imaging system (SOMATOM Definition, SIEMENS) |
| Liao NQ et al 2024 | 2 | Siemens Somaton Sensation 64- slice MSCT and GE Discovery 750 HD CT scanners |
| Vithayathil M et al 2025 | 4 | Siemens, GE Healthcare, Phillips, Toshiba |
| Xu XN et al 2025 | 1 | 8-channel phased-array body coil-equipped Siemens 3.0 T Prisma MR scanner |
| Xu J et al 2025 | 7 | GE LightSpeed VCT, GE Revolution CT, PHILIPS Brilliance 64, GE Discovery CT750, SIEMENS 49, SOMATOM Definition Flash, or GE Optima CT680 |
| Lu DY et al 2025 | 6 | uMR586/680 (United Imaging Healthcare, Shanghai, China); Aera/Avanto/Symphony Tim (Siemens Healthcare, Erlangen, Germany); Signa HDxt/HDe/Voyager (GE Healthcare, Milwaukee, WI, USA); Prisma/Skyra (Siemens Healthcare, Erlangen, Germany); Discovery MR750/Signa HDxt (GE Healthcare, Milwaukee, WI, USA); Achieva/Ingenia ElitionX (Philips Medical Systems, Best, Netherlands) |
| Yin LN et al 2025 | 2 | GE (Optima) 64-slice spiral CT or Siemens (SOMATOM Definition Flash) dual-source spiral CT |
| Zhu YM et al 2025 | 3 | MAGNETOM Prisma 3.0T (Siemens), Discovery MR750 3.0T (GE Healthcare) and Signa HDx 1.5T (GE Healthcare) |
| Ding GY et al 2025 | 1 | 64-slice spiral CT (General Electric Company, America) |

**Supplementary Table S5**. Clinical factors integrated into clinical-radiomics models.

| Study | Predicted outcome | Clinical factors |
|---|---|---|
| **ICIs** | | |
| Yuan GS et al | Treatment response (mRECIST) | tumor embolus, ALBI grade |
| **ICIs combined with molecular targeted therapy** | | |
| Xu B et al | Treatment response (RECIST1.1) | extrahepatic disease, HBV DNA, AFP, macrovascular invasion, sum of diameter of baseline intrahepatic target lesions |
| Dong W et al | OS | ALBI, tumor diameter, ALT, and portal vein invasion |
| Vithayathil M et al | OS, PFS | BCLC stage, MVI, extrahepatic metastases and PLR |
| Xu XN et al | PFS | BMI, tumor size, albumin |
| Xu J et al | OS, PFS | Up-to-seven criteria, lung metastasis (LungMet), AFP level, and tumor size |
| **ICIs combined with molecular targeted therapy and local therapy** | | |
| Lu DY et al | Treatment response (mRECIST) | TACE type |
| Yin LN et al | Treatment response (mRECIST), PFS | AFP, VP4 type portal vein tumor thrombus, tumor diameter, presence of capsule, diffuse tumor type |
| Ding GY et al | OS | Albumin-bilirubin (ALBI) grade and portal vein tumor thrombus (PVTT) |
